# Brazilian Citizens: Expectations Regarding Dairy Cattle Welfare and Awareness of Contentious Practices

**DOI:** 10.3390/ani7120089

**Published:** 2017-11-26

**Authors:** Clarissa S. Cardoso, Marina A. G. von Keyserlingk, Maria José Hötzel

**Affiliations:** 1 Laboratório de Etologia Aplicada e Bem-Estar Animal, Departamento de Zootecnia e Desenvolvimento Rural, Universidade Federal de Santa Catarina, Florianópolis 88034-001, Brazil; clarissa.cardoso@gmail.com; 2 Animal Welfare Program, Faculty of Land and Food Systems, The University of British Columbia, Vancouver, BC V6T 1Z4, Canada; nina@mail.ubc.ca

**Keywords:** animal welfare, survey, ideal farm, cow-calf separation, zero-grazing, disposal of male calves, dehorning

## Abstract

**Simple Summary:**

Animal welfare is an important issue for citizens in North America and Europe, but much less is known about how citizens from emergent countries, such as Brazil, view this topic. Our aim was to explore attitudes of urban Brazilian citizens about dairy production and, in particular, how they view four routine husbandry practices: early cow-calf separation; zero-grazing; culling of the newborn male calf; and dehorning without pain mitigation. Through in-depth interviews and a questionnaire using open-ended questions, we can conclude that animal welfare was a major issue for our participants, especially in terms of its perceived relation with milk quality. Although participants were initially unaware about any of the four management practices, they were all viewed as contentious and not supported. This study provides some insights that farmers and others working in the Brazilian dairy supply chain should take into consideration, particularly in terms of social sustainability.

**Abstract:**

The primary aim of this study was to explore attitudes of urban Brazilian citizens about dairy production. A secondary aim was to determine their knowledge and attitudes about four potentially contentious routine dairy cattle management practices: early cow-calf separation; zero-grazing; culling of newborn male calves; and dehorning without pain mitigation. To address the first aim 40 participants were interviewed using open-ended semi-structured questions designed to probe their views and attitudes about dairy production in Brazil, and 300 participants answered a questionnaire that included an open-ended question about the welfare of dairy cattle. Primary concerns reported by the participants centered on milk quality, which included the rejection of any chemical additives, but also animal welfare, environmental and social issues. The interviewees rarely mentioned animal welfare directly but, when probed, expressed several concerns related to this topic. In particular, participants commented on factors that they perceived to influence milk quality, such as good animal health, feeding, clean facilities, and the need to avoid or reduce the use of drugs, hormones and pesticides, the avoidance of pain, frustration and suffering, and the ability of the animals to perform natural behaviors. To address our second aim, participants were asked questions about the four routine management practices. Although they self-reported being largely unaware of these practices, the majority of the participants rejected these practices outright. These data provide insight that animal welfare may be an important issue for members of the public. Failure to consider this information may increase the risk that certain dairy production practices may not be socially sustainable once lay citizens become aware of them.

## 1. Introduction

Over the past 50 years, animal advocacy groups and concerned citizens have increasingly questioned intensive animal production practices [1]. In some countries this has led to regulations governing how animals must be cared for on farms (e.g., New Zealand, the European Union), while in other countries, particularly the United States (US), corporations have exerted pressure for improved changes [2,3,4]. For example, US retailers such as McDonald’s and Walmart announced that after 2015 they will no longer source eggs from farms that use cages [5,6]. The United Egg Producers in the US also recently announced that they would eliminate the practice of euthanizing male chicks [7]. The German Parliament has also stated that they will no longer allow male chick to be euthanized immediately following hatch [8,9]. The European Union has also announced that surgical castration of pigs without anesthesia will be phased out by 2018 [10]. Similar changes have been seen in other parts of the world, for example, in Brazil, where existing regulations do not specify mandatory standards of care for specific farm management practices [11]. Large processors in Brazil including Aurora, BRF and JBS have announced that they are phasing out sow’ gestation crates [12,13,14]. This demand for change has resulted in Brazilian animal agricultural industries having to rethink their practices.

Given that these changes can also create upheaval for the farmers, it is important that we continue to try to understand how the public views farming practices. Clearly, if we are able to anticipate practices that may be potentially contentious, then we can arguably pursue sustainable options that work for the farmers, the animals and resonate with societal values [15]. Failure to do so can result in situations where the public may boycott products or support legislation that sets out to ban certain practices, but that may not necessarily work for the farmers or the animals under their care. Public acceptance is an essential component of the sustainability of the food animal industries [16,17].

Little is known about the views of citizens from developing countries, such as Brazil, regarding animal production systems [18], specifically if animal welfare is an important issue for them when considering specific animal production systems. Bonamigo et al. [19] showed that despite Brazilian consumers initially not being concerned about the welfare of broilers, they reversed their position once they were informed about how chickens are raised for meat. Similarly, Souza et al. [20] reported that participants changed their perception about meat consumption after seeing images regarding mistreatment of farm animals. Brazilian citizens were also shown to prefer free-range, cage-free, and more natural production systems when asked their views about different production practices used to raise laying hens, beef cattle, pregnant and lactating sows, and poultry meat [21]. Finally, the majority of Brazilian citizens surveyed by Hötzel et al. [22] rejected two common practices used in the dairy industry, zero-grazing and cow-calf separation, for animal welfare reasons. Indeed, if animal agriculture industries are to remain sustainable in the long run, they must work towards implementing practices that resonate with societal values.

Our overall aim was to explore attitudes of Brazilian urban citizens about dairy farming. Using a convenience sample we performed two studies, both designed to gain insights into what Brazilians viewed to be an ideal dairy farm. A secondary aim was to assess awareness and acceptability of four potentially contentious husbandry practices routinely used in dairy production.

## 2. Materials and Methods 

This study used a mixed methods approach, which combines collection and analysis of quantitative and qualitative data with the aim to provide greater understanding of a research problem than would be possible when using only a single method [23]. The first part of this work involved in-depth interviews that made use of open-ended semi-structured questions designed to allow for a grounded theory approach to elicit the participants’ views and attitudes about dairy production in Brazil. The second part made use of a questionnaire that had an open-ended question that allowed the participant to convey their views on dairy cattle welfare. This was followed by multiple quantitative questions designed to determine the participant’s awareness about specific dairy production practices. The study was exploratory in nature, with the overall aim to gather information that could be used to generate more refined hypotheses for further investigations, and thus use non-probabilistic samples [24]. 

We used a convenience sample of Brazilian urban citizens that were 18 years or older, self-identified as not being involved in dairy production, and balanced for gender. The number of participants interviewed (*n* = 40) was based on the criterion that the sample size must be sufficient for the responses obtained to provide content diversity and richness regarding the issue of the study, and that the addition of more participants does not increase the diversity or richness of the sample, referred to as saturation [25]. For the questionnaire our goal was to collect information from a sufficiently diverse group of participants. Our sample size of 300 for the questionnaire was based in part on previous work [22] that collectively mirrored a representative sample of the population living in the south region of Brazil [26].

The Ethics Committee of Research on Humans of the Federal University of Santa Catarina, Brazil—protocol number 1,195,546 and 1,538,926—approved both studies. All research participants were recruited at the Hercílio Luz International Airport, in Florianópolis, Brazil, given that this location facilitates an intense movement of people. People waiting for flights or passengers in the public area before security in the airport lounge were invited to voluntarily participate in the study. People who self reported as not being involved in dairy production, and who agreed to participate after they had been told that we would need about 15 min of their time, were invited to participate in the study.

### 2.1. Study 1: In-Depth Interviews

Immediately after approaching a potential participant the researcher asked whether they were interested in participating in a research study that would take about 15 minutes. If a positive answer was obtained, the researcher went through the consent form with the participant and asked for signed consent before the interview was initiated. With the exception of four of the 40 interviewees, all interviews were audio recorded. In the case of the four participants consent was not given to be audio recorded, but they did consent to the interviewer taking detailed notes about the conversation. The interview started with a single open question: *How do you imagine an ideal dairy farm?* The participants typically started by simply describing what they viewed to be the characteristics of an ideal dairy farm, but then the researcher used open-ended questions to elicit the reasons why the respondent considered these characteristics important. At the end of the interview the researcher asked the following demographic questions: age, sex, education, area of residence, and region of Brazil. This frequently stimulated further conversation that was then used to explore specific issues that the researcher felt needed clarification. The interview ended with a final question where the interviewer asked the participant if they had any further comments. The lead author transcribed the interviews for use in the subsequent thematic analyses.

### 2.2. Study 2: Questionnaire

Participants (*n* = 300) were informed of the purpose of the research, and presented with a printed consent form. Upon signing the consent form, and after participants were assured that their participation would remain anonymous, they were given the questionnaire. The same demographic questions as outlined above for the in-person interviews were also asked in the questionnaire, followed by an open-ended question: *In previous research done by our team, some people have told us that an ideal dairy farm should pay attention, among other things, to the welfare of their animals. If you agree with that statement, can you tell us what you would expect of a dairy farm that takes care of the welfare of their animals? Consider, please, the cows and their calves*. On a separate page, and after answering the open-ended question, participants were invited to answer closed questions structured to determine whether they were aware of (yes/no) and position (support to oppose) using a five point Likert scale, regarding four routine management practices used on dairy farms. The specific practices were presented in simple, short sentences, as follows: cow-calf separation, *the newborn dairy calf is separated from its mother shortly after birth*; zero-grazing, *on some dairy farms cows are reared inside barns, without access to pasture at anytime when lactating*; culling the newborn male calf, *some male calves are killed immediately after birth because they are not used to produce milk*; dehorning/disbudding, *the horns of young calves are removed without use of any medication to control the pain*. The order of the practices was randomized among participants. Open-ended responses were transcribed into a digital document by the lead author.

### 2.3. Analyses

To address our first objective, the interviews and responses to the open ended question in the questionnaire were analyzed according to the methodology outlined by Huberman and Miles [27], which consists of coding the information, identifying themes, and organizing the information to allow for the drawing of conclusions. For the responses of the open-ended questionnaire question we used 228 responses, having excluded those where the responses were illegible. The first and last authors coded all of the qualitative data, and an invited third expert in qualitative analyses independently examined 10 and 30 of the interviews and questionnaires, respectively. These three readers compared results and reconciled any discrepancies. Quotes presented in this document were translated to English by the first and last authors and back translated to Portuguese by an invited researcher to verify that the original meaning was preserved.

To address our second objective quantitative data were analyzed descriptively through basic statistics, using means and percentages. From an initial sample of 300 respondents, four were excluded from the analysis because they declared some involvement with dairy production, leaving 296 usable responses. The five point Likert scale question about the position of participants regarding the four practices was reclassified into three points (support/indifferent/oppose).

## 3. Results

By design we did not include any participants in both studies that self identified as having some involvement in dairy production. We also intentionally targeted individuals such that our group of participants in both studies were balanced for gender, and were similar in terms of representing the age classes in the latest Brazilian census [26]. The majority was urban, from the south and southeast regions, and was highly educated, with nearly 90% holding a bachelor degree (Table 1). Although we did not ask participants specifically about their income, a recent report released by the Brazilian Civil Aviation Office [28] based on a representative sample of Brazilians indicates that the majority of domestic air travelers within Brazil are middle class, and approximately 20% of the upper class.

### 3.1. Qualitative Findings

#### 3.1.1. Study 1: Interviews—Features of an Ideal Dairy Farm and Their Associated Reasons

Interviewees’ main concerns revolved around assurances of high milk quality and absence of chemical additives to the milk, both justified as important considerations for people’s health. Interviewees also said that on an ideal farm, animals should not suffer, and that the environment and the employees should be respected. The ideal dairy farm should also be profitable, the main reasons being farmers’ quality of life and the country’s economy.

Regarding milk quality, interviewees described the ideal farm as a place that must respect hygiene and be clean. There was an expectation that the milk be produced without the use of added hormones and antibiotics: *as few chemicals as possible* (S1–32); *Antibiotics are always harmful to health, not only for the people but also for the animal* (S1–11). Interviewees also talked about the role of animal feed in the context of milk quality. Most interviewees mentioned feed quality, considered pasture or natural feed as the ideal, e.g., *I believe that ideally cows should eat natural things—that would be pasture. Natural would be better because I believe that the cow would not necessarily produce more milk, but would produce better milk* (S1–7). However, one interviewee associated concentrate with better milk quality, i.e., *Confinement, concentrate, are good for milk quality* (S1–5). Some also related animal health to milk quality, e.g., *Because if the animals are not well the product won’t be as good as it could be* (S1–17). Absence of agrochemicals and transgenic components in the pasture was also mentioned in relation to milk quality, e.g., *Agrochemicals are used to produce pasture and this goes into the meat and milk. They should be used in a way that doesn’t harm humans* (S1–18).

There was a high expectation by the interviewees that there be an absence of animal suffering, i.e., *No suffering for the animals at milking* (S1–9); *With animal welfare, animal comfort; let’s say a happier animal, something like that* (S1–26), and that animals should be *well cared* or *well reared*, which could mean *well fed*, *good space*, *freedom* (S1–6). Individuals also expressed their desire that animals be provided enough space: *Animals with adequate space, because I saw that some of them are tied and I think this is outrageous because nobody likes to be confined. Nobody was made to be confined, not a bird, not a cow, not anybody, not us, nobody likes it. And I think a cow mustn’t be happy there* (S1–11); *It is shocking that cows are not on pasture they must be free, because like it or not, it is an aggression towards the animal; she works the whole day, no time off, this is an aggression towards the animal, I think* (S1–6). For some interviewees, the way animals are treated results in better milk quality, e.g., *If the animal has a good quality of life, the product will be better* (S1–20). In this context, it was mentioned that employees should be trained, and family farms were identified as the best option, because the farmers are in the best position to care for the animals: *because the (family) farmers live closest to the animals and provide them with humane treatment, like giving them a name; this treatment, I think, also influences the product* (S1–2). The use of hormones was also related to the animal’s health, *Because I think they harm the animal’s health. I think they cause tumors or something. I do not see them as a good thing* (S1–40).

Naturalness was also an important characteristic raised by the interviewees; many were concerned with the animals’ feed, and pasture-based systems were considered ideal because of their naturalness, e.g., *many cows on pasture because its natural its closer to nature, they can feel better* (S1–10); *natural food as it was at the beginning of Creation, green pasture* (S1–29). Some showed negative attitudes towards modernity and technology in terms of equipment, and related these features to loss of naturalness: *Something more manual and not so much machinery (about milking)* (S1–2); *I don’t think it’s necessary (machinery), it’s not good because it’s not natural* (S1–10); *I think anything that is too industrialized brings some harm to our health. I think this kind of milk is too pasteurized. I think the best is natural milk, as more natural is better. More natural milk, even if it lasts less, it is better* (S1–26). A few interviewees associated the lack of naturalness, the use or presence of chemicals, and recent diseases to milk consumption: *I remember when I was a kid, my mother used to say that milk was a very healthy food. And today it isn’t anymore. So what happened between then compared to today for milk not be healthy anymore? Because you see information that milk has fat, this and that and that today everyone is allergic to lactose. So, I've been wondering what has happened. Could it be that in our time the way milk was produced was healthier than it is today?* (S1–31); when reflecting to the importance of chemicals added in the milk, the same interviewee concluded: *Why not using the most natural way possible? We know that’s good, because it’s natural* (S1–31).

Profit was another major concern raised by the interviewees. They discussed the importance of profit for farmers’ livelihoods and way of life, the country’s economy, and the economic sustainability of the industry: *Ideally, a farm should not need subsidies* (S1–3). Some mentioned that if a farm is profitable farmers might generate employment. The export of milk products and production of special products, for example without lactose was also discussed. Aspects of animal management, including cows’ access to water, good feeding, health and housing, genetics, and even music in the milking parlor, were discussed in the context of increased production and profit.

Some interviewees commented that they would like to have more knowledge about how animals are reared, and a few of them commented on their sources of information, e.g., *These days I have read that animals suffer a lot* (S1–40) and cited sources where they gathered information such as television and the Internet. Many started the interview saying they did not know anything about dairy farming, and for this reason they had no idea how an ideal farm should be, but as the interview progressed they did convey, to some degree, what they expected in a dairy farm. For example, one individual began by asking a question and then immediately went on to answer: *The animals are confined, right? I think I agree more or less with this way. I don’t know if it could be much better. It could be but I can’t imagine how. Maybe with animals not confined in such small spaces, something like that* (S1–25). Others revealed that they did not like what they imagined a dairy farm to be: *Everyone knows it’s a real production line* (S1–2); *Back in the day the cows produced milk without concentrates, today there are concentrates, but we don’t know what they are made of. Some say that they have chemical additives, others say they don’t, I don’t know to what extent this is true because we are in an environment with lots of information and we don’t know what information is correct* (S1–31).

Many interviewees declared some discontent towards the dairy industry, saying that they did not know whether people still drank milk or what the industry added to the milk, i.e., *I don’t know which sorts of preservatives are put into the milk to allow it to stay in contact with aluminum. Lots of studies say that aluminum is not ideal for food preservation. Because aluminum is carcinogenic* (S1–7); or that the industry is not truthful in its presentation of the image of dairy farming: *Confined, I suppose that’s how it is. I think it is a utopic, naive thing to think that cows are kept as you see in the ads or on the box of milk, a cow’s picture on pasture, free and happy... I don’t think so* (S1–40). One interviewee said that because of this, *consumers prefer not to think about the origin of the product that they consume* (S1–2). Many interviewees talked about government inspection of commercialized products, e.g., *I think there should be more government oversight of the milk trade. The milk leaves the farm and we don’t known how it is preserved, how the storage places are* (S1–7), usually recalling recent cases of milk adulteration in Brazil: *Some of my friends do not drink milk from some companies, and others do not drink milk at all, because of the (cases of milk) adulteration that have been reported a lot lately* (S1–14).

#### 3.1.2. Study 2: Questionnaire—Important Aspects Related to Animal Welfare on a Dairy Farm

The 228 responses collectively resulted in 4994 words (on average each response was 22) that were then coded into four themes (Table 2). Many responses bridged more than one theme and were thus coded into multiple themes. This means the examples given below, many times bridge more than one theme.

Questionnaire participants were primarily concerned about the feed given to the cows, e.g., *Adequate feeding for the cows* (S2–88); *Healthy feeding for the animals* (S2–103); *Nutritious pastures for milking cows* (S2–191). Some participants provided more general responses calling for adequate animal facilities, while others were more specific, citing hygiene and cleanness of facilities: *Adequate housing* (S2–182); *with excellent hygiene conditions* (S2–19). Good health and veterinary care were also cited, e.g., *Animal health care* (S2–158). Participants also mentioned access to drinking water and shade, thermal comfort, and access to shelter: *Natural shade, if possible, and high quality water available* (S2–1); *(The farm) must have a place for animals to protect themselves from the sun and rain, where they can sleep and eat, and provided adequate veterinary care* (S2–7)*; Adequate thermal conditions* (S2–31).

Most participants associated animal welfare with the quality of treatment given to animals or, in their words, *ethical management* (S2–75). Participants expressed concern regarding animal mistreatment and, in their opinion, animals should receive adequate management, should be treated well, and mentioned *quality of life*, *being careful with the animals, avoiding aggression and mistreatment* (S2–18); *Adequate and decent dairy cows’ management* (S2–14); *I’d hope all animals are cared for with the same level of care as humans* (S2–22). Some expected audits to ensure that handlers treated animals well, e.g., *In my opinion there should be a more rigorous oversight and animals should be treated the best possible way* (S2–2).

For many participants, good quality treatment results in more production, e.g., *Adequate treatment to animals, without mistreatment, which also results in better product quality and productivity* (S2–30); *All animals must be respected and loved, so their production increases* (S2–28), and in better product quality: *I hope animals are well cared for, so the products have better quality* (S2–167) and consumers’ health: *Cows’ welfare is reflected in product quality, with evident consequences for our health* (S2–23).

Additionally, some participants related the ability of animals to express natural behaviors, e.g., *With animals free within nature* (S2–47); *Animals that live with freedom* (S2–122); *Not limiting animals’ freedom. allowing a normal life cycle*. (S2–213); *Pastures free of pesticides, as natural as possible* (S2–183); *That cows were not forced to give birth constantly to sustain milk production* (S2–47). Other respondents commented about the way animals are reared; that cows should graze in large, open spaces, or should be fed pasture and more natural, less industrialized, or organic feed, e.g., *I hope animals to be grass-fed, not grain-fed* (S2–157); *Cows reared on pasture* (S2–200). Some participants specifically rejected indoor housing, e.g., *Animals should not be confined* (S2–32); *Not under radical confinement; some freedom to move and the possibility to graze* (S2–109).

Concerns about natural production were associated with criticisms regarding excessive or inadequate use of chemical products, e.g., *Pasture without pesticides, as natural as possible* (S2–183); *Without artificial stimuli to increase production* (S2–19); *Without products to accelerate growth* (S2–201); *I’d expect them feed the animals without pesticides and hormones* (S2–151); *A farm that offers organic feed to its animals* (S2–114); *To avoid too many medicaments* (S2–170). One reason for this rejection was the perception that some of these substances could affect the health of animals, e.g., *The use of veterinary phytotherapic drugs, to avoid contaminating the animals* (S2–14), but also the consumers: *The volume of chemicals present in the milk cause health problems to consumers* (S2–185).

Some participants also mentioned the emotional lives of the animals, stating that they should live without stress, frustration, suffering, or pain, e.g., *Never inflicting pain to animals* (S2–27); *There should be at least anesthesia to remove the horns* (S2–149); *I’m not in favor of any kind of mistreatment and managements that cause pain or suffering* (S2–50). Also animals should be happy and comfortable, e.g., *A well cared for animal is a happy animal* (S2–46), including playing music for the cows, e.g., *Using classic music during milking* (S2–6). A few participants (5% of total of participants) mentioned early cow-calf separation, e.g., *That cows and calves be allowed to live together* (S2–51). Some argued that the cow and calf should spend at least some time together, or that farmers should be more careful with calves because the cows are supposed to produce milk for the calves.

#### 3.1.3. Study 2: Questionnaire—Knowledge of Specific Dairy Farming Practices

Regarding how informed the questionnaire participants considered themselves to be about dairy production, 2% said very informed, 24% somewhat informed, 13% intermediate, 27% somewhat uninformed, and 34% totally uninformed. In the open responses 10% of the participants addressed their own lack of knowledge of the proposed topic. For example, some said they had never thought about a dairy farm or the welfare of the animals or had no idea how a dairy farm works but nonetheless most expressed several opinions, e.g., *I totally ignore how a dairy farm works, but I believe that it should be designed to benefit all animals, environment and humans* (S2–13); *As I’m not expert in the subject, I don’t know what resources are needed, but as for my concern, it is with the volume of chemicals inside milk that cause health problems to the population* (S2–185); *I don’t know how a dairy farm works, but I believe that all efforts must be made so that the animals don't suffer or feel pain; there must be quality of life* (S2–221).

### 3.2. Quantitative Findings

#### Study 2: Questionnaire—Awareness and Support (Or Not) for Contentious Practices

Most participants answered that they were not aware of the four described practices commonly used on dairy farms. However, once they were made aware of the practices, most rejected them (Table 3). Awareness of the specific practices was also low: early cow-calf separation (45%), zero-grazing (32%), culling the newborn male calf (21%), and dehorning/disbudding without pain control (15%). Those that were aware cited the following sources as vehicles of information: Internet (26%); TV (21%); friends or family (16%); printed material such as newspapers, magazines or books (11%); a visit to a farm (9%); rural upbringing or having lived in a rural area (7%); animal protection societies (5%); personal experience (5%); and school (1%).

## 4. Discussion

This study provides insights about the knowledge and expectations of Brazilian lay citizens regarding what an ideal dairy farm is, and the welfare of dairy cattle living on Brazilian dairy farms. Most participants were unaware of the four specific practices that are commonly done on dairy farms; more importantly, once they became aware they overwhelmingly rejected these practices. Although interviewees described an ideal dairy farm and were in many cases not knowledgeable about dairy farming, they did cite characteristics related to a farm as a whole such as milk quality. Many also discussed animal welfare as an ethical requirement, and that profitability of the dairy farm was essential for social reasons.

Not surprising given the knowledge base that the participants had about dairy farming, they frequently mentioned animal welfare as a broad concept but rarely explored specific aspects about dairy farming that they thought influenced animal welfare. However, when probed specifically about animal welfare the participants in our study brought up similar issues and concerns as other citizens from other countries [18,29], with the majority of their comments focusing on at least one of the three common constructs of animal welfare, affective states, biological functioning and naturalness [30].

Concerns relating to biological functioning and health are frequently raised as the most important aspect of animal welfare by farmers and veterinarians [31,32,33] but, interestingly, our participants in both studies expressed similar concerns, desiring that the animals’ basic needs such as feeding and health, clean, comfortable and appropriate animal facilities should all be part of an ideal dairy farm. The participants related these concerns as important given their perceived effects on milk quality, also shown by others [22,34,35]. Other aspects of herd management that our participants perceived would influence milk quality and consequently people’s health, such as the type of feed, medicines and chemical additives used for milk production, were also mentioned. These concerns are supported by many reports on the potential down stream effects on human health from residues found in food and water, including agrochemicals (e.g., [36]), hormones (e.g., [37,38]) and antibiotics (e.g., reviewed by [39]). Given the discussion in the public domain in many countries about the misuse of antibiotics in human and animal health in agriculture, and its relation to antibiotic resistance [40], it is not surprising that our participants raised this topic. The search for healthy food to prevent diseases in humans is a contemporary concern around the world that is endorsed by the scientific community (e.g., [41,42]). Concerns about the impact of additives to animal feed or antibiotics have been reported as one of the motivations driving people to buy organic food [43]. Despite the interest in organic production systems and products shown by our participants and in other studies [35,44,45,46], access to organic animal agriculture products may be limited in some emerging countries [47], given challenges associated with both technical support, and sourcing animal feed that is free of transgenic components [48].

Naturalness, another concern expressed by many participants in both studies as they discussed animal welfare, was expressed in terms of preference for production systems that allow for natural behaviors such as grazing, and access to space. This may be driven in part by the on-going discussion regarding restriction of movement in the pig and egg industries, which have both received considerable attention in the public domain in Brazil [21]. The desire for more naturalness has been reported to be rooted in beliefs and values held by lay citizens of different countries [22,35,45,49,50]. For consumers, naturalness in terms of food likely has a variety of meanings, including tasty, fresh and healthy food, food containing no chemical residues, food containing natural ingredients, and food that has been minimally processed or processed under homemade, organic, local, or eco-friendly systems [51].

Some participants expressed a desire for traditional, less industrialized farms. This rejection of technology in dairy production systems may be explained by an association with problems identified concerning the welfare of other domesticated animals such as pigs and chickens, rural communities and the environment, brought by industrialization of animal production and confinement systems [52]. Indeed, lay citizens often reject aspects of these so called industrialized systems, such as fertilization treatments [53] and zero-grazing [45]. The desire for a *return to the past* suggested by some participants of this study has been discussed by Fraser [52] as a romantic view of agriculture, an expectation that a return to models of agriculture used before industrialization would solve the ethical problems of modern animal production. However, not all people appear to hold this view. For example, Cardoso et al. [35] reported that, when asked, American millennial respondents described an ideal dairy farm as being modern and made use of technologies as important tools needed for efficiency and, in turn, profitability. There is also a growing body of evidence that there is a strong desire by the public that farming embrace sustainability [49,54], and thus technologies that are socially acceptable may be play a role in the future [17,55].

Many participants in both studies discussed the ethical treatment of animals as good quality of treatment, absence of pain, suffering and stress. This was somewhat surprising given that most identified themselves as being largely uninformed about dairy farming. However, this may be explained at least in part by the growing presence of animal advocacy campaigns exposing contentious practices in farm animal production systems, including dairy production, and the repeated replication of the issues raised in these campaigns in television programs and social media in Brazil [56,57,58,59]. Respondents listed the Internet, TV, friends and family as main sources of information, giving some support to this conclusion.

Across both studies more than half of the participants self identified as having little or no knowledge about dairy farming, an issue also raised by many of the participants in their responses. Their lack of knowledge was also apparent in the words they used to describe their ideal dairy farm, with descriptors such as *properly* or *adequate* frequently used, rather than specific terms or references to specific practices and their outcomes. Not surprisingly given their background, most participants had low awareness of the four common dairy management practices described in the questionnaire [60,61,62,63]. Despite the low awareness of the existence and prevalence of these practices on Brazilian dairy farms, participants overwhelmingly opposed them, a phenomenon also shown in other surveys [22,64,65,66]. Others have also shown that citizens have little or no knowledge of how farm animals are reared but, when asked, consider animal welfare highly important and show interest in knowing more about the issue [67,68].

Some have argued that the agricultural industries must place greater efforts in educating citizens about farming practices as a means to improve understanding and acceptance of some contentious practices [33,53,69,70]. However, there is a growing body of work indicating that when people gain knowledge about the practices, it does not lead to increased acceptance, but rather it decreases confidence about whether farmers are able to provide their animals a reasonably good life [22,64,66,71]. These findings reiterate that lack of knowledge of livestock farming does not explain the low support for practices perceived to reduce animal welfare. Engagement of the public in the development of new technologies, in contrast, may be one way to make the dairy industry more socially sustainable [15,72].

The ideal type of production system was also discussed by many participants, with some rejecting indoor housing and concentrate feeding, while others describing an ideal farm as one with open spaces and animals grazing on pasture. Most interesting is that in both of these later arguments participants cited naturalness and animal welfare when justifying these characteristics. Some have argued that pasture access for dairy cattle provides benefits for animal health and welfare, while others have argued that indoor housing reduces dairy cattle health and the ability of the animals to express natural behaviors (reviewed by [73]).

There is tremendous potential within Brazil to produce pasture-based milk, given the favorable climatic conditions throughout much of the country [1]. Interestingly, a study discussing possible future scenarios for the milk supply chain in Brazil by 2020 identified animal health, food safety, and environmental issues as the major challenges facing the industry [74]. These challenges, along with animal welfare, should be taken into consideration throughout the milk supply chain, as failure to do so may result in risks given the lack of trust expressed by many of our interviewees. 

Small farms, which are largely family run, have an important role in the Brazilian milk supply chain because they produce a substantial amount of the total milk produced in the country [75]. If the dairy industry is negatively affected due to the existence of animal welfare concerns, thousands of family farmers that make their livelihoods from dairy sales could potentially be affected [75]. When well managed, pasture-based systems are profitable [76,77] and may help the social sustainability of dairy farming.

The convenience sample used in this study should not be considered representative of the Brazilian population, given that the higher level of education of the respondents and the distribution within regions of Brazil was not representative. However, the findings herein do represent the ideas of a group of mixed sex, urban, well-educated middle class Brazilian citizens that spanned a variety of ages. Although having a higher socio-economic status can result in greater emphasis being placed on animal welfare regulations by citizens, it was not an aim of the present study to elucidate the effects of socio-economic status on attitudes and views pertaining to farm animal welfare. The methods used in the current study assumed that citizens drive changes on food production, not consumers (see Aerts [78]). Moreover, more educated people, not necessarily with high incomes, tend to be more concerned about animal welfare than those that are not aware of the issue [18]. Over the last decades the socio-economic status of the Brazilian population has improved and animal welfare, as well other contemporary issues, has increasingly played a greater part of citizens’ daily life. 

## 5. Conclusions

When the participants were invited to imagine a dairy farm they showed a special concern for milk quality, but also mentioned the social importance of dairy farming and animal welfare. The participants that were prompted to give their opinions about animal welfare made associations with the quality of cows’ treatment (avoidance of pain, frustration and suffering), good animal health, feeding and hygiene, the ability of the animals to perform natural behaviors, and the need to avoid or reduce the use of drugs, hormones and pesticides. The majority of participants in this study were unaware of farming practices of early cow-calf separation, zero-grazing system, the culling of newborn male calf and dehorning/disbudding without pain mitigation, but when made aware overwhelmingly rejected these practices. These findings suggest that participants of this study were mostly unaware about common animal production practices but were highly concerned about milk quality and animal welfare. These studies provide insight that animal welfare is indeed important to some members of the public, as was the case for our group of highly educated urban middle class Brazilians, and there is risk that certain routine dairy production practices may undermine the socially sustainability of this industry once the public becomes aware of them.

## Figures and Tables

**Table 1:** Demographics of participants separated by interviews and questionnaires.

Demographics	Variable	% Participants Interview (*n* = 40)	% Participants Questionnaire (*n* = 296)
Age (years)	18–24	7	26
	25–34	42	35
	35–44	18	17
	45–54	10	11
	55 or above	23	11
Sex	Male	48	44
	Female	52	56
Level of education	High school	10	22
	Some college	2	7
	Bachelor’s degree	81	48
	Graduate degree	7	23
Area of residence	Urban	98	98
	Rural	0	1
	Suburban	2	1
Region of Brazil	North	2.5	6
	Northeast	7.5	4
	Centre-West	17.5	11
	Southeast	32.5	33
	South	40	46

**Table 2:** Emerging themes in response to the question, *In previous research done by our team, some people have told us that an ideal dairy farm should pay attention, among other things, to the welfare of their animals. If you agree with that statement, can you tell us what would you expect of a dairy farm that takes care of the welfare of their animals?* (*n* = 228 participants).

Theme	*n* ^1^	(%) ^2^
Biological functioning	108	47
Quality of animal treatment	86	36
Naturalness	50	22
Affective states	44	19
Total ^3^	288	124

^1^ Number of references codified into each theme; ^2^ Percent relative to number of references in relation to total arising from all participants; ^3^ Total exceeds 228 participants and 100% as some responses were sometimes coded into multiple themes.

**Table 3:** The percentage of participants (*n* = 296) who were unaware, and who then either rejected, were indifferent or supported early cow-calf separation, zero-grazing systems for dairy cows, culling the newborn male calf, or disbudding/dehorning calves without pain control once they were informed about the practice.

Topic	Unaware (%)	Reject (%)	Indifferent (%)	Support (%)
Early cow-calf separation	65	84	14	2
Zero-grazing	68	85	13	2
Culling the newborn male calf	79	90	9	1
Dehorning without pain control	85	89	10	1
